# Phytates as a natural source for health promotion: A critical evaluation of clinical trials

**DOI:** 10.3389/fchem.2023.1174109

**Published:** 2023-04-14

**Authors:** Sónia M. G. Pires, Rita Silva Reis, Susana M. Cardoso, Raffaele Pezzani, Esteban Paredes-Osses, Ainur Seilkhan, Alibek Ydyrys, Miquel Martorell, Eda Sönmez Gürer, William N. Setzer, Ahmad Faizal Abdull Razis, Babagana Modu, Daniela Calina, Javad Sharifi-Rad

**Affiliations:** ^1^ LAQV-REQUIMTE, Department of Chemistry, University of Aveiro, Aveiro, Portugal; ^2^ Phytotherapy Lab (PhT-Lab), Endocrinology Unit, Department of Medicine (DIMED), University of Padova, Padova, Italy; ^3^ AIROB, Associazione Italiana per la Ricerca Oncologica di Base, Padova, Italy; ^4^ Instituto de Ciencias Naturales, Facultad de Medicina Veterinaria y Agronomía, Universidad de Las Américas, Las Américas, Chile; ^5^ Educational Program, Geography, Environment and Service Sector, Abai Kazakh National Pedagogical University, Almaty, Kazakhstan; ^6^ Biomedical Research Centre, Al-Farabi Kazakh National University, Almaty, Kazakhstan; ^7^ Centre for Healthy Living, Department of Nutrition and Dietetics, Faculty of Pharmacy, University of Concepción, Concepción, Chile; ^8^ Unidad de Desarrollo Tecnológico, UDT, Universidad de Concepción, Concepción, Chile; ^9^ Department of Pharmacognosy, Faculty of Pharmacy, Sivas Cumhuriyet University, Sivas, Türkiye; ^10^ Aromatic Plant Research Center, Lehi, UT, United States; ^11^ Department of Chemistry, University of Alabama in Huntsville, Huntsville, AL, United States; ^12^ Department of Food Science, Faculty of Food Science and Technology, Universiti Putra Malaysia, Serdang, Selangor, Malaysia; ^13^ Natural Medicines and Products Research Laboratory, Institute of Bioscience, Universiti Putra Malaysia, Serdang, Selangor, Malaysia; ^14^ Department of Biochemistry, Faculty of Science, University of Maiduguri, Maiduguri, Borno, Nigeria; ^15^ Department of Clinical Pharmacy, University of Medicine and Pharmacy of Craiova, Craiova, Romania; ^16^ Facultad de Medicina, Universidad del Azuay, Cuenca, Ecuador

**Keywords:** phytate, nutrients, health benefits, clinical trials, organophosphorus compound

## Abstract

Phytates are a type of organophosphorus compound produced in terrestrial ecosystems by plants. In plant feeds, phytic acid and its salt form, phytate, account for 60%–80% of total phosphorus. Because phytate is a polyanionic molecule, it can chelate positively charged cations such as calcium, iron, and zinc. Due to its prevalence in vegetal tissues and the fact that people consume plants, phytate was first considered a potential health benefit. This updated review aims to summarize the current data on the results of clinical trials of phytates on human health, highlighting both beneficial and undesirable effects. To obtain these updated data, published papers in electronic databases such as PubMed/MedLine, TRIP database, Wiley, Google Scholar, Baidu, and Scopus were searched. Study results have shown that phytate can have beneficial health effects such as antioxidant, anticancer potential and reduction of pathological calcifications in blood vessels and organs; but also, negative effects by reducing the absorption of minerals important for maintaining the homeostasis of the human body. According to these recent results derived from recent clinical studies, phytates may be a potential natural source for health benefits. To improve clinical efficacy and human health benefits, further dose-response studies are needed to determine effective therapeutic doses and potential interactions with conventional drugs.

## 1 Introduction

Phytates [chemically known as myoinositol (1,2,3,4,5,6) hexakisphosphate] can be found in numerous plants and their parts, including seeds, nuts, legumes, and cereals (Habtamu Fekadu, 2014; Pujol et al., 2023). Phytates possess the trivial names of inositol hexakisphosphate (IP6, InsP6) or phytic acid, with the last one commonly used. Phytates in plants represent a source of energy and antioxidant capacity (as a phosphate groups donor), but they have a predominant role as storage mineral chelating agents (depot of Cu^2+^ and Zn^2+^ cations in particular) due to the presence of negative charges at physiological pH (Barrientos and Murthy, 1996; Persson et al., 1998; Raboy, 2003). In addition, this compound is present outside the plant kingdom and can be found in all organisms (excluding prokaryotes), where, together with its derivatives, it is involved in cell metabolism, homeostasis, and pathological conditions (Bohn et al., 2008; Maffucci and Falasca, 2020; Silva et al., 2021). Phytates were first discovered in 1855 by a German researcher Theodor Hartig who identified circular particles in different plant seeds (Hartig, 1855; Khoo and Wolf, 1970). The chemical structure of phytate was later defined in 1914, as myo-inositol-1,2,3,4,5,6-hexakis dihydrogen phosphate (Anderson, 1914). The resulting insoluble salts denominated as phytates consist of a ring with six carbon atoms esterified with a phosphate group (IP6). Phytases promote IP6 dephosphorylation into smaller inositol phosphates (IP1-IP5) also belonging to the phytates family. Given its abundance in vegetal tissues and the consumption of plants by humans, phytate was early investigated for a possible role in health (Schlemmer et al., 2009). Indeed, already in the 1940s phytate was suggested as an anti-nutrient, due to its capacity to couple with cations and reduce absorption (McCance and Widdowson, 1942; Mc and Walsham, 1948). Subsequently, it has been reported that a large number of phytates in an unbalanced diet could represent an issue, however in a normal diet with low phytate content, phytates can be considered safe and not implicated in nutrient deficiency (Lopez et al., 2002). More recently the interaction between phytates and human diet and health has been revised suggesting numerous potential beneficial effects (Schlemmer et al., 2009). For example, phytate has been implicated in preventing calcifications (Grases and Costa-Bauza, 1999; Fernandez-Palomeque et al., 2015), in diabetes (Omoruyi et al., 2013; Sanchis et al., 2018), in lipid profile (Koba et al., 2003; Yuangklang et al., 2005), in a healthy diet (Prieto et al., 2010), in cancer (Vucenik and Shamsuddin, 2006; Pallem et al., 2020; Markiewicz et al., 2021), and in dental medicine (Parkinson et al., 2018; Liu and Tu, 2021). Non-etheless, a few works reported the potential negative impact of phytate (Kim et al., 2020; Kim et al., 2021), while clinical trials experimenting with phytates in human diseases are poorly represented (Hanson et al., 2006; Hunt and Beiseigel, 2009; Prieto et al., 2010; Sanchis et al., 2018). This review highlights the pros and cons of phytates, with a special emphasis on the therapeutic health beneficial effects in humans.

## 2 Review methodology

Relevant information on the results of clinical trials of the beneficial effects of phytates on human health was collected by searching scientific databases such as PubMed/MedLine, TRIP database, Wiley, Google Scholar and Scopus using the next MeSH terms: “Humans,” “Kidney Failure,” “Chronic,” “Phytic Acid,” “Renal Dialysis,” “Vascular Calcification,” “Antioxidants/metabolism,” “Apoptosis/drug effects,” “Cell Proliferation/drug effects,” “Dietary Supplements,” “Humans,” “Phytic Acid/pharmacology,” “Calcinosis/drug therapy,” “Calcinosis/pathology,” “Calcium, Crystallization,” “Phytic Acid/therapeutic use.” There were evaluated 70 articles from international publications that included pharmacological studies with identified mechanisms of action and clinical studies of phytates regarding the effects in various chronic diseases. Of the studies reviewed, only papers written in English were selected. Abstracts, short communications, experimental pharmacological studies that did not include mechanisms of action, or that used homeopathic medicines were excluded. We also explored https://clinicaltrials.gov/website to identify clinical trials with phytates. The most relevant data have been summarized in tables and illustrative figures. The chemical formulas have been validated according to PubChem (PubChem, 2022).

## 3 Bioavailability and pharmacokinetics data

**FIGURE 1:**
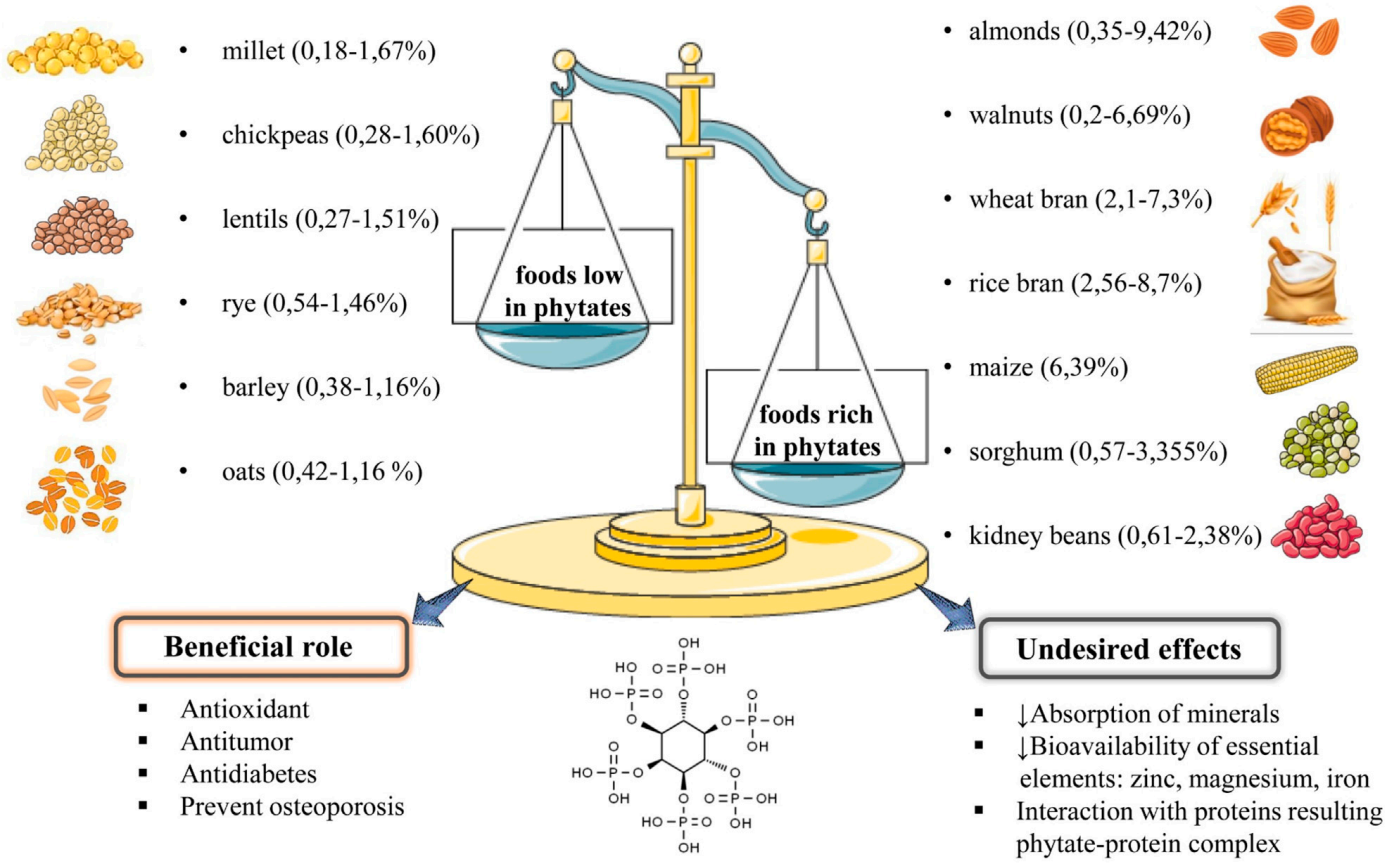
Summarized diagram regarding the main sources of phytates, their quantities in foods, beneficial role and undesired effects.

For several decades, phytic acid was considered an anti-nutrient due to its ability to form insoluble salts with cations such as zinc, magnesium, iron, and copper, which could cause a decrease in the bioavailability of these essential elements. In the last 20 years, several studies have shown that this effect is more evident in cases of a diet very rich in phytates, combined with a deficiency in trace elements (Vucenik and Shamsuddin, 2003; Silva and Bracarense, 2016). However, as the bioactive properties of phytate have been elucidated over the years, namely, its antioxidant and antitumor capabilities, the understanding of its digestion and absorption processes, as well as their interactions with the components of the human body, has sparked a lot of interest. Phytate is usually found in high quantities in almonds (0.35%–9.42%) (Harland and Oberleas, 1986), walnuts (0.2%–6.69%) (Chen, 2004), wheat bran (2.1%–7.3%) (Harland and Oberleas, 1986), maize (6.39%) (Kortekangas et al., 2020), rice bran (2.56%–8.7%) (Kortekangas et al., 2020), sorghum 0.57%–3.355% (Kortekangas et al., 2020), kidney beans (0.61%–2.38%) (Lehrfeld, 1994), soybeans (1%–2.22%) (Gupta et al., 2015); and in small quantities in other vegetables and seeds: millet (0.18%–1.67%) (Ravindran et al., 1994), chickpeas (0.28%–1.60%) (Ravindran et al., 1994), lentils (0.27%–1.51%), rye (0.54%–1.46%) (Gupta et al., 2015), barley 0.38%–1.16% (Kortekangas et al., 2020); oat 0.42%–1.16% (Harland and Oberleas, 1986); (Figure 1), albeit it can be destroyed during food processing like soaking and malting.

**FIGURE 2:**
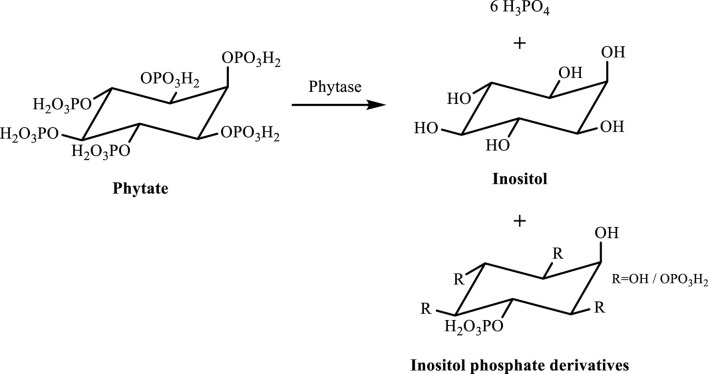
Phytate hydrolysis in inositol, inositol phosphate derivatives, and orthophosphate (Lei and Porres, 2003).

This is usually hydrolyzed to inositol, inositol phosphate derivatives, and orthophosphate (Figure 2) by phytases found in microorganisms or plants. Plant intrinsic phytases, on the other hand, can only be triggered under specific conditions, such as cooking, fermentation, and immersion (Schlemmer et al., 2009). Thus, in general, when an ingested food is high in phytates, this is hydrolyzed mostly in the intestines by microbial phytases (Weremko et al., 1997). Complexed cations and proteins are also released when phytases cleave the phosphate groups from the phytate, enhancing their bioaccessibility.

In the past, being negatively charged at physiological pH, phytate and its main derivatives were assumed not to permeate the lipid bilayer of plasma membranes, and therefore, their absorption was highly questionable. This paradigm, however, has changed over the years (Nahapetian and Young, 1980), as subsequent studies have suggested the absorption of its derivatives, mainly in the intestines. Among them, Nahapetian and Young (1980) used radioactively labelled ^14^C of myo-inositol and discovered that this was detected in rats’ urine, blood, and internal organs. Accordingly, Grases et al. (2001) demonstrated that a normal diet rich in phytates, or the adding it supplements to a normal human diet, resulted in increased inositol hexaphosphate (IP6) levels in plasma and excreted in urine. Moreover, in a clinical trial, Grases et al. (2006a) also reported that, independently of stomach conditions, 8 h after intake of 400 mg of phytates followed by a low-phytate diet resulted in an accumulation of more than 400 µg of IP6 in the urine excretion. Note that, according to Dinicola et al. (2017), these compounds are rapidly digested and distributed throughout the body following oral consumption, with their derivatives reaching their peak plasma levels within 4 h. Still, regardless of these data, it is unclear whether phytate derivatives absorption is mediated by pinocytosis, active transporters, or other processes. So far, in a study aimed at investigating the anti-cancer mechanism of IP6, the authors reported that IP6 entered HeLa cells via pinocytosis (Ferry et al., 2002). It is also known that phytates can interact with proteins due to the affinity of phosphate groups for cationic amino acids. This interaction can be harmful, disrupting protein digestion, but it can also protect the human body from harmful effects of specific proteins, such as oxidases and pathogenic proteins and several diseases like diabetes. Lee et al. (2006) found that diabetic KK mice fed with a diet supplemented with 0.5% and 1.0% sodium phytate for 8 weeks *ad libitum*, induced a decrease in blood glucose levels. Because phytate may bind to some amino acids and inhibit digestive enzymes, the low glycemic response could be related to the delayed digestion of starch (Lee et al., 2006). Affinity competition, pH, pI, ionic strength, and amino acid availability are all variables that can alter the stability of these complexes. These can be manipulated to prevent phytate-protein complex formation (Wang and Guo, 2021).

## 4 Phytate in clinical trials

**FIGURE 3:**
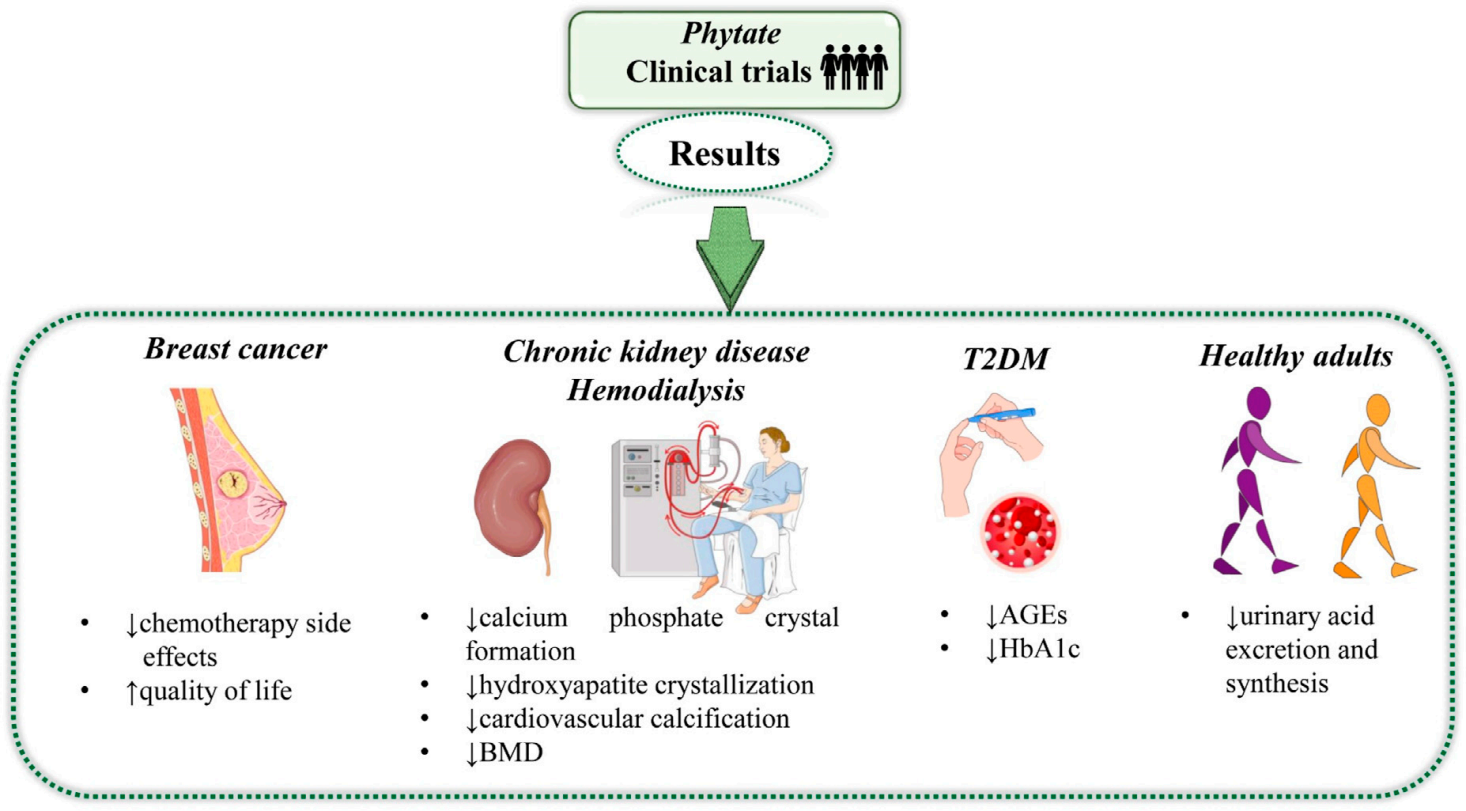
Summarized diagram with the most relevant results of natural and artificial forms of phytate. Abbreviations and symbols: ↓decrease, ↑increase, AGEs, glycation end-products and HbA1c, glycated hemoglobin.

Phytate is a natural plant-derived compound, however artificial forms of this compound are also marketed. The effect of natural (plant-derived) and artificial forms has been studied on different diseases, for instance, breast cancer, T2DM, cardiovascular calcification, and BMD (Tables 1, 2; Figure 3). For reviewing clinical trials about phytate, studies published in English found in the ClinicalTrials^®^ and PubMed^®^ databases in the last 5 years have been collected. In a double-blind, randomized, clinical trial, Proietti et al. (2017) investigated the effect of phytate on 20 adult breast cancer patients. The patients were separated into two groups. The treatment group was administered phytate (200 mg) per day and the control group received a gel containing hyaluronic acid (5 g) per day for 6 months. At the end of the 6 months, questionnaires belonging to the “European organization for testing the treatment of cancer (EORTC)” consisting of several questions under different parameters were requested from the patients. Patients’ assessments of the questionnaires showed that phytate administration not only increased the life quality and ability to perform daily life activities but also decreased adverse effects caused by chemotherapy in the treatment group compared to the control group. The level of glycation end-products (AGEs) and glycated hemoglobin (HbA1c) are higher in T2DM patients and the approach for the attenuation in the level of these proteins or ligands could be promising in the management of this disease (Quispe et al., 2022). A cross-over, randomized, clinical trial conducted by Sanchis et al. (2018) reported that a phytate-based diet could be used for this purpose. In the study, 35 adult T2DM patients were divided into phytate-based diet groups and non-phytate-based diet groups. The phytate-based diet group was administered 380 mg of phytate, three times per day for 12 weeks. After that, 12 weeks wash-out period was given to each group. At the end of the wash-out period, groups were switched to the alternate diet for 12 weeks. Observations on the patients were continued for another 12 weeks. The outcome of the study showed that the levels of AGEs and HbA1c were diminished after phytate-based diet intervention compared to the beginning. In another double-blind, randomized, crossover, clinical study, Ikenaga et al. (2020) showed the effect of phytate on serum uric acid (SUA) levels in healthy volunteers. 48 healthy volunteers were chosen and divided into a phytic acid drink (which contains 600 mg of phytic acid) ingested and a control drink (in case, mineral water) ingested groups. Results indicated that the level of SUA was decreased in the phytic acid drink ingested group compared to the control group. The outcome of the study showed that phytic acid has the potential to improve life quality by regulating the SUA level.

**TABLE 1:** Clinical trials with natural form of phytate in the last 5 years.

References	Design	Treatment	Patients	Results
Proietti et al. (2017)	Clinical trialRandomizedDouble-blind reference-controlled	Phytic acid(200 mg)/dayDuration: 6 months	AdultBreast Cancer*n* = 20	-Phytate administration decreased the side effects of chemotherapy and increased the quality of life
Sanchis et al. (2018)	Clinical trialRandomizedCrossoverOpen study	Phytate(3 mg × 380 mg)/dayDuration: 3 months	AdultT2DM*n* = 35	-The level of AGEs and HbA1c was lower in the IP6 diet group compared to the non-IP6 dieted group-No adverse effect
Ikenaga et al. (2020)	Clinical trialRandomizedDouble-blind Crossover	Rice-derived phytic acid drink(50 mL)/visitDuration: -	AdultHealthy*n* = 48	-Urinary acid excretion and urinary acid synthesis decreased in the phytic acid drink-infused group compared to the control group-No adverse effect

AGEs, glycation end-products; HbA1c, glycated hemoglobin; T2DM, type 2 Diabetes Mellitus.

**TABLE 2:** Clinical trials with an artificial form of the phytate in the last 5 years.

References	Design	Treatment	Patients	Key effects
Perelló et al. (2018)	Clinical trialRandomizedDouble-blindplacebo-controlled	SNF472(2 mg/kg× 9 mg/kg)/weekDuration: 4 weeks	AdultHD*n* = 8	-Calcium phosphate crystal formation was lower in the plasma samples of HD patients receiving the SNF472 compared to the placebo control
Salcedo et al. (2019)	Clinical trialRandomizedDouble-blindplacebo-controlled	SNF472(3 × 1 10 mg/kg)/weekDuration: 5 weeks	AdultHD*n* = 8	-The hydroxyapatite crystallization was decreased significantly in the treatment group compared to the control group-No adverse effect
Raggi et al. (2020)	Clinical trialDouble-blindPlacebo-controlled	SNF472(3 mg× 300 mg, 600 mg)/weekDuration: 52 weeks	AdultRenal Disease*n* = 274	-The progression of coronary artery and aortic valve calcification in patients was attenuated by SNF472 as compared to the placebo-controlled group
Bellasi et al. (2021)	Clinical TrialRandomizedDouble-blindPlacebo-controlled	SNF472	Adult	-Cardiovascular calcification was reduced in the SNF472 treated group as compared to the placebo-control
Bushinsky et al. (2021)	Clinical TrialRandomizedDouble-blindPlacebo-controlled	SNF472(3 mg × 300 mg, 600 mg)/weekDuration: 52 weeks	AdultHD*n* = 274	- BMD was decreased in all groups, especially in the 600 mg SNF472 group at the end of 52 weeks
Dold et al. (2020)	Clinical trialRandomizedSingle-blind	FeSO4, FePP and Fe-PA-HCP(1 mg × 4.2 mg)/dayDuration: 33 days	AdultHealthy*n* = 23	-Fe-PA-HCP, co-ingested with bouillon, has a promising effect on patients with iron-depleted women

BMD, bone mineral density; HD, hemodialyses; BMD, bone mineral density.

Mainly two different artificial forms of phytate SNF472 and Fe-Pa-HCP have been extensively studied on cardiovascular calcification levels of hemodialysis patients or patients with renal disease at different stages. A randomized, double-blind, placebo-controlled clinical study by Perelló et al. (2018) investigated the effect of SNF72 on cardiovascular calcification. In the study, the effect of SNF472 was initially analyzed on 16 healthy volunteers with different doses (0.5, 5, 9, 12 mg/kg) for 4-h infusions. Pharmacokinetics (PK) analysis showed that in maximum SNF472 concentration measured in plasma (Cmax) and area under the curve (AUC) parameters increased in a dose-dependent manner but not significantly. Additionally, treatment-emergent adverse events (TEAEs) were observed during the treatment period. At the end of PK and TEAE analyses, the effective dose was determined at the Dose Escalation Safety Committee (DESC) meeting as 9 mg/kg. The chosen effective dose was applied to eight hemodialysis (HD) patients. Patients were assigned into two groups. One group was infused with 9 mg/kg of SNF472 and the other with a placebo for 4 h during their dialysis session. The drugs were infused into the patients two times per week for 4 weeks. Results of the study revealed that calcium phosphate crystal formation which is associated with cardiovascular calcification was significantly lower in the SNF472 administered group compared to the placebo control. Another randomized, double-blind, placebo-controlled, clinical trial with different doses of SNF472 was also conducted by the same research group to test the effect of SNF472 on HD patients and its inhibitory effect on HAP crystallization. SNF472 with several doses (1, 3, 5, 12.5, and 20 mg/kg) were administered by patients three times a week. At the end of the treatment period, pharmacokinetics analyses in the study showed no significant difference in the Cmax and AUC parameters. Additionally, SNF472 administration did not cause any adverse effects on HD patients. To determine the effect of SNF472 on cardiovascular calcification in HD patients the effective dose was selected as 10 mg/kg by DESC. Then, HD patients were administered the determined effective dose of SNF472 or placebo three times per week for 4 weeks. At the end of 4 weeks, the level of HAP crystallization was decreased in the SNF472-administered group compared to the placebo-controlled (Sanchis et al., 2018). A clinical trial performed by Raggi et al. (2020) examined the effect of SNF472 on cardiovascular calcification in patients with end-stage renal disease. In this randomized, placebo-controlled, double-blind, phase 2b study, 274 patients aged 18–80 years were selected and divided into three groups: placebo, SNF472 (300 mg), and SNF472 (600 mg) for 52 weeks. According to the study, SNF472 was administered to the patients three times a week after hemodialysis. The results showed that aortic valve progression and coronary artery calcification were less in the SNF472 (600 mg) group compared to the placebo-controlled group. The same research group also designed the CaLIPSO (Cal for calcium and ipso meaning the item itself) trial which is a multicenter, double-blind, placebo-controlled, randomized clinical study to evaluate the effect of SNF472 on hemodialysis patients with cardiovascular calcification in the coronary arteries, aorta and aortic valve. In the study, the calcium levels of the patients were initially screened for 28 days. At the end of the screening period, 274 patients were chosen depending on their Agatston coronary artery calcium (CAC) score (101–399) and randomized in a 1:1:1 ratio to administer placebo, SNF472 300 mg or SNF472 600 mg three times per week for 52 weeks. At the end of 52 weeks, the CAC score of the HD patients was significantly reduced in the SNF472-treated groups as compared to the placebo-controlled group (Bellasi et al., 2021). In general, HD patients have a low BMD, which could be associated with cardiovascular calcification (Orlic et al., 2010; Kim et al., 2021). Therefore, any dietary intervention that increases BMD could be a promising approach for the management of this problem in HD patients. Regarding this, in a further study by the same group, Bushinsky et al. (2021) investigated the effect of the SNF472 on BMD in HD patients with Agatston CAC score (101–399) which were tested with a CaLIPSO trial. The design of this study was the same as the previous one. The outcome of this study showed that SNF472 treatment did not increase the total hip and femoral neck BMD in HD patients. The total-hip BMD was significantly reduced in the 600-mg SNF472-treated group compared with the placebo control. Overall, these studies indicated that SNF472 was effective in reducing cardiovascular calcification, however, it did not have an effect against BMD in HD patients. Recently, the effects of phytate on iron absorption have been investigated by using three different ferrous derivatives. In a controlled, single-blind, randomized crossover trial, 23 adult healthy women were administered the above-mentioned derivatives over 33 days. The iron absorption of the substances was investigated with two different enhancers, cornmeal, and bouillon. According to the randomly allocated groups, two different fortifiers, namely, bouillon, and cornmeal were administered to the patients, as well as Fe-Pa-HCP (ferric iron complexed with phytic acid and hydrolyzed corn protein), FePP (ferric pyrophosphate) and FeSO_4_ (ferrous sulfate). Especially, Fe-Pa-Hcp given together with bouillon showed a promising effect on the patient with iron deficiency (Dold et al., 2020).

Overall, all these studies showed that both natural and artificial forms of the phytate have a promising effect on healthy volunteers, breast cancer, hemodialysis patients and cardiovascular calcification. None or minor adverse effects were reported in the aforementioned clinical trials. However, we should note that all clinical trials have some limitations such as a low number of patients, short intervention time, etc. So, further clinical trials were needed to deeply understand the positive or negative effects of phytate administration.

## 5 Discussion and therapeutic perspective

**FIGURE 4:**
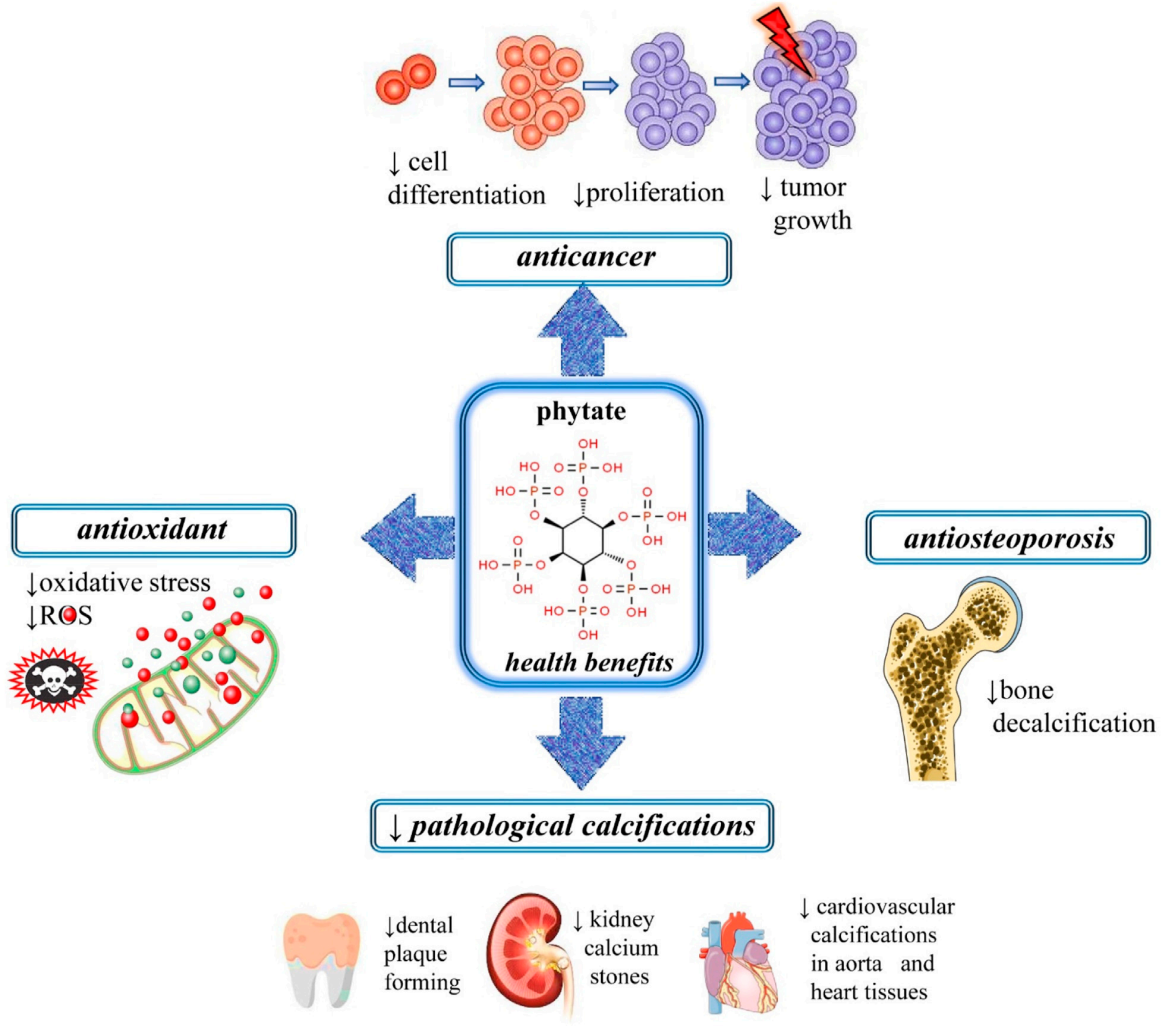
Illustrative figure with the most important therapeutic applications of phytates. Abbreviations and symbols: ↑increase, ↓decrease, ROS, reactive oxygen species.

It is already established that phytate levels found in mammals are strongly dependent on dietary intake (oral or topical), rather than on endogenous synthesis. Besides being a naturally occurring antioxidant, dietary phytates have been claimed to prevent kidney stone formation, protect against diabetes mellitus, caries, atherosclerosis and coronary heart disease, as well as to fight the progression of a wide range of cancers (Abdulwaliyu et al., 2019; Bloot et al., 2021; Kumar et al., 2021) (Figures 3, 4).

### 5.1 Phytate as antioxidant

Oxidative stress occurs as a result of an imbalance between free radicals and antioxidants found in our body (Calina et al., 2016; Salehi et al., 2019; Sharifi-Rad et al., 2021a; Sharifi-Rad et al., 2021c). Free radicals can be formed from dioxygen molecules, which contain unpaired electrons (Calina et al., 2016; Islam et al., 2021; Alshehri et al., 2022; Ozkan et al., 2023). It is precisely because of the unpaired electrons that oxygen molecules react easily with other molecules (Tsoukalas et al., 2019a; Tsoukalas et al., 2019b; Salehi et al., 2019; Sharifi-Rad et al., 2021b; Salehi et al., 2021). This results in chemical chain reactions (called oxidation reactions) that can have beneficial or harmful consequences (Calina et al., 2020; Mititelu et al., 2020; Islam et al., 2021; Amin et al., 2022). Due to their chelating ability and antioxidant activity many compounds can act as regulators of heme iron excess, avoiding oxygen-initiated free radical formation and, ultimately, preventing damaging Fenton reactions (Nussbaum et al., 2017; Buga et al., 2019; Calina et al., 2020; Bhuia et al., 2023). Furthermore, inositol hexaphosphate (IP6) also proved to be effective in reducing the occurrence of metal-catalyzed protein glycation, such as advanced glycation end products (AGEs), which may trigger diabetes-related diseases (López-Moreno et al., 2022). The antioxidant capacity of phytates was first demonstrated by Sanchis et al. (2018), in chemical models, as well as in a randomized crossover study of 33 patients with type II diabetes mellitus (T2DM). Notably, the authors concluded that the dietary supplementation with IP6 for 3 months resulted in lowering the levels of circulating AGEs (∼25%) and glycated hemoglobin (3.8%) in the patients (Sanchis et al., 2018; Omoruyi et al., 2020).

### 5.2 Protective effect of phytate against pathological calcifications

Like other polyphosphates, phytates are considered to be powerful inhibitors of calcium salt crystallization in urine and soft tissues. Moreover, they show high-affinity binding to the calcium of hydroxyapatite (HAP) crystals through chemisorption, avoiding both crystallization and redissolution processes. Besides protecting against pathological calcifications (e.g., dental calculi, renal calculi and cardiovascular calcifications), they also present beneficial effects in the treatment of the bone decalcification process occurring during osteoporosis (López-González et al., 2008; Sanchis et al., 2021). The benefit of IP6 on bone decalcification associated with osteoporosis was demonstrated by Lopez-Gonzalez et al. (2019), in a study performed with 143 women through monitoring of bone mineral density (BMD) in the lumbar column and the neck of the femur, and questionnaires regarding osteoporosis risk factors. Regardless of the limitation of the volunteer group (inclusion only of women with a maximum of 5 years since menopause), the authors could conclude that the phytate consumption by postmenopausal women reduced their risks of hip and major osteoporotic fractures, being the positive effect more pronounced in women with risk factors of osteoporosis associated (Lopez-Gonzalez et al., 2019). The ability of IP6 in inhibiting brushite and HAP crystallization was demonstrated by Grases et al. (2009) in a study performed with 29 healthy dental plaque-forming volunteers. The subjects rinsed their mouths for 1 min, twice each day, with mouthwash solution containing phytate (0.142% as a percentage by weight) and zinc, having concluded that the treatment with zinc phytate significantly inhibited the development of tartar formation (about 70%) (Grases et al., 2009). Several studies put in evidence the ability of phytate to inhibit oxalate monohydrate formation and calcium phosphate crystals (Grases and Costa-Bauzá, 1999). The potential of IP6 in the treatment of renal calculi was also demonstrated (Grases and Costa-Bauza, 2019). As an example, in the work of Curhan et al. (2004), the authors concluded that rats fed with an AIN-76A diet (purified rodent diet containing no phytate) developed mineral deposits at the kidney corticomedullary junction, whereas a complete absence was observed in a group fed with AIN-76A + 1% IP6. In a prospective study including 96,245 females, aged 25–42, the regular ingestion of dietary IP6 was shown to significantly reduce the risk of calcium stones (Curhan et al., 2004). The women included in the study had no history of kidney stones and were followed for an 8-year period, and their diets were accessed with self-administered food frequency questionnaires. Regarding the preventive effects on cardiovascular calcifications, Grases et al. 2008, Grases et al. 2006b) reported that the oral supplementation of Wistar rats with a balanced diet (UAR-A04) containing phytate, for 76 weeks, reduced calcium deposition in the aorta and heart tissues. A retrospective cross-sectional study performed by Sanchis et al. (2016) referring to 69 chronic kidney disease patients, demonstrated that the adequate consumption of phytate could efficiently prevent abdominal aortic calcification. The phytate consumption levels were inferred from 10 food-frequency questionnaires validated on the same day of the 2-h urine analysis. In another study; Fernández-Palomeque et al. (2015) evaluated the relationship between physiological levels of urinary phytate and heart valve calcification, in a population of 188 elderly subjects (mean age: 68 years). The valve calcification of the subjects was measured by echocardiography and urinary phytate was also assessed. In addition, blood samples were also collected as part of each medical history to ascertain the existence of concomitant diseases, cardiovascular risk factors, medication usage and food habits. The obtained results clearly showed an inverse correlation between urinary phytate content and mitral annulus calcification, thus suggesting that the consumption of phytate-rich food may help to prevent cardiovascular calcification evolution (Fernández-Palomeque et al. (2015). The effects of oral supplementation with different nutrients (magnesium, zinc, iron, vitamin K) including phytate were also recently assessed in patients with calcific aortic valve stenosis but the results of the study, despite promise (Vucenik, 2019), have not yet been fully disclosed (Donato et al., 2021).

### 5.3 Anticancer potential

The onset of cancer is due to the uncontrolled multiplication and mutation of body cells that eventually form a tumor (Tsoukalas et al., 2019a; Ianoși et al., 2019; Jain et al., 2021; Salehi et al., 2021; Motyka et al., 2023). Several pharmacological experiments demonstrated the anticancer effects of phytate. Distinct studies support the hypothesis that IP6 reduces cell proliferation, and induces apoptosis and differentiation of malignant cells with reversion to normal phenotype, affecting several critical molecular targets (Vucenik, 2019). A broad spectrum of laboratory data has been accumulated over more than 30 years since the pioneering assays reported by Shamsuddin and Ullah, 1989, 1988), who reported the great antineoplastic potential of IP6 in rat models involving cancer of the large intestine, treated with Na-IP6 drinking water. In addition to confirming IP6 as a preeminent broad-spectrum antineoplastic agent, additional experiments also demonstrated the preventive and therapeutic potential of IP6 against cancers of different cells and different tissue systems. For example, de Lima et al. (2015) demonstrated the cytotoxic effects of a phytic acid nickel complex, on human acute leukemia Jurkat T cells. The dephosphorylated metabolites (IP4 and IP5) of phytate also presented anticancer activity and remarkably proved to be more potent in inducing apoptosis of HeLa cells than phytate (Ferry et al., 2002). Phytate anticancer effects were also assessed against several other cancer cell lines: breast and prostate cancer; cervical cancer; HepG2 hepatoma; rhabdomyosarcoma, for which it was demonstrated the efficient inhibitory effect of IP6 in human malignant cell growth in a dose and time-dependent manner (Vucenik and Shamsuddin, 2006). Other pharmacological experiments also put in evidence of phytate antineoplastic properties not only for colon cancer but also for breast carcinoma, skin papillomas, liver cancer or metastatic fibrosarcoma. The animal experiments, in agreement with *in-vivo* assays, highlighted a significant decrease in the number and size of tumors. Furthermore, the synergistic anticancer activity of phytate when combined with inositol was also observed (Vucenik and Shamsuddin, 2006; Vucenik, 2019). Encouraging results come also from pilot clinical studies in advanced colorectal cancer with multiple liver and lung metastases. IP6 was given to 22 patients given as an adjuvant to chemotherapy and an overall reduction of tumor growth was noticed. Furthermore, in some cases a regression of the neoplastic lesions was also detected (Vucenik, 2019). The use of IP6 in combination with chemotherapy was shown to reduce side effects, allowing the patients to perform their daily activities (Proietti et al., 2017). A case reported by Khurana et al. (2019) on a patient with metastatic melanoma, who refused the conventional chemotherapy treatment to be treated only with IP6 + inositol supplement, achieved complete remission of cancer and remained in remission 3 years later (Khurana et al., 2019).

## 6 Conclusion

In this review, we have compiled information on the pharmacological potential, bioavailability, clinical trials, and advantages and disadvantages of phytates, with a focus on therapeutic health effects in humans. The role of phytates in human health has been described as a dual problem since their discovery. On one hand, studies have shown that phytates can reduce the bioavailability of essential minerals for maintaining the homeostasis of the human body; on the other hand, they have proven beneficial effects for human health, such as antioxidant, the reduction of side effects of chemotherapy or the significant reduction of hydroxyapatite crystallization in hemodialysis patients. Future research in preclinical studies is needed to elucidate more pharmacological mechanisms of action of phytates as well as adverse effects and potential drug interactions. Translational pharmacological studies to determine the exact therapeutic dose effective in humans are also needed.
